# Dynamical anomalous transport of molecules subject to inhomogeneous body forces

**DOI:** 10.1007/s00033-025-02614-7

**Published:** 2025-12-01

**Authors:** A. Girelli, G. Giantesio, A. Musesti, R. Penta

**Affiliations:** 1 Dipartimento di Matematica e Fisica “N. Tartaglia”, Università Cattolica del Sacro Cuore, via della Garzetta 48, 25133 Brescia, Italy; 2 “Department of Numerical Analysis and Scientific Computing (SCAN)”, Simula Research Laboratories, Oslo, Norway; 3 “Mathematics for Technology, Medicine & Biosciences”, Università degli Studi di Ferrara, Ferrara, Italy; 4 School of Mathematics and Statistics, University of Glasgow, Glasgow, UK

**Keywords:** Advection–diffusion equation, Homogenization, Multiple time scales, Molecules transport, 35Q92, 92B05, 35B27

## Abstract

This work explores diffusion with scale-dependent coefficients, starting from a general advection–diffusion framework from a theoretical standpoint, and then focusing numerically on a purely diffusive regime. Advection–diffusion processes are central to modeling transport phenomena in natural and engineered systems. However, classical models often fail to capture the complexities of systems with spatial and temporal variability. In this work, we present a multiscale advection–diffusion model that incorporates time-dependent diffusion coefficients and spatially inhomogeneous, multiscale body forces. Using the asymptotic homogenization technique, we derive a macroscopic equation that reflects the evolution of transport properties across multiple scales, accounting for both spatial and temporal variations. A key contribution of this study is the formulation of new cell problems associated with the dual time-dependence of the diffusion coefficient and the multiscale forces, which lead to the introduction of additional source terms. Furthermore, we incorporate a novel source term arising from the nonzero divergence of the advective velocity field, which modifies the effective macroscopic advection velocity to capture source and sink effects at the microscale. We apply this model to describe water molecule diffusion in packed erythrocytes, a system exhibiting dual time scales, and by showing how our approach captures the temporal evolution of transport under dynamic diffusion.

## Introduction

This study stems from the intention to investigate the diffusion process with a diffusion coefficient that depends on two spatial and temporal scales. We first analyze the problem within a broader theoretical framework by examining the advection–diffusion equation, and then focus on a numerical study of the purely diffusive case. Advection-diffusion processes are key to modeling transport phenomena in diverse natural and engineered environments. While the classical diffusion equation captures the random spread of quantities such as particles or energy, real-world systems often involve transport (advection) in addition to diffusion. The advection–diffusion equation offers a more accurate representation by incorporating both effects. However, in many complex systems, neither the diffusivity nor the advective velocity remains constant. Instead, both can vary across space and evolve over time due to environmental changes, feedback mechanisms, or system dynamics. To reflect this, modern modeling efforts must go beyond traditional assumptions and develop multiscale frameworks that incorporate time-dependent diffusion coefficients and time-varying velocity fields. These advanced models better capture the intricate behavior of transport processes across multiple temporal and spatial scales, leading to deeper insights into the underlying physics of heterogeneous media.

A multiscale advection–diffusion equation with a time-dependent diffusion coefficient and a time-dependent velocity describes phenomena where the rate of diffusion and velocity changes over time, and this can happen due to various causes. As for the diffusion coefficient, it can depend on the temperature or on the concentration itself, or diffusion rates can vary with metabolic cycles or environmental fluctuations [2, 10, 40, 44, 46, 49, 59, 62, 64, 65, 68, 69]. Moreover, in lymph nodes and tumors the spreading of drugs or the movement of immunological cells may also depend on time, reflecting changes in the microenvironment or cellular activity [27, 50]. Incorporating a time-dependent diffusion coefficient enables a more accurate model of these temporal changes, allowing for predictions that align more closely with experimental data and observed phenomena.

Multiscale forces play a fundamental role in governing the behavior of heterogeneous materials, particularly in the context of porous media subjected to external body forces. These forces operate at different spatial and temporal scales, influencing macroscopic properties such as flow, diffusion, and material response. The study of their effects is crucial for understanding and predicting the behavior of complex systems, including biological tissues, fractured rocks, and engineered materials. Inspired by the work of [4, 21, 56], in which asymptotic homogenization is applied to derive effective governing equations for porous media under the action of inhomogeneous body forces, this paper extends the aforementioned paper [56] by considering homogenization in different spatial and temporal scales.

Moreover, the macroscopic model introduced in this work features a novel source term arising from the nonzero divergence of the velocity field. This additional term alters the standard macroscopic advection component by capturing microscale source and sink effects, which are often overlooked in classical formulations. The inclusion of this term not only enriches the physical interpretation of the model but also requires the formulation of a new cell problem to properly account for its influence within the multiscale framework.

In this paper, we focus on the formulation and multiscale analysis of an advection–diffusion equation with a time-dependent diffusion coefficient, a time-dependent multiscale velocity and multiscale forces using the asymptotic homogenization technique. In particular, we use this technique to describe an advection–diffusion equation that presents inhomogeneity in space and time, obtaining a macroscopic equation averaging the inhomogeneity of both space and time variables. As far as we know, this is the first attempt to homogenize both in space and time using a formal homogenization technique, taking into account both the microscale time during the homogenization process, multiscale forces and multiscale velocities that depend on both space and time: indeed, in [43, 47, 48] two different time scales are taken into account, but then the diffusion process is not supposed to depend on the time scale associated with the microscale length, a natural hypothesis that raises from the homogenization technique. In [6, 7, 17, 57, 58], the authors consider two time scales in homogenization but do not consider spatial scale separation, and microscale time variations are eventually smoothed out by means of a microscopic time average. In our case, as the diffusion coefficient depends on the microscale time as well, we cannot assume that the diffusion process does not depend on the time scale associated with the microscale length. The dependence of the diffusion coefficient on the microscale time leads to variations in the microscale problem which reflect on the macroscopic diffusion coefficient. In [18, 34] the authors consider a diffusion problem with the diffusion coefficient that depends on both microscopic and macroscopic space and time: our model is an extension of the latter works considering an advection–diffusion equation with multiscale forces and multiscale velocities with nonzero divergence that depend on both microscopic and macroscopic space and time. Moreover, in this work we propose numerical simulations of the microscopic cell problem and compare the results with the classical steady diffusion problem.

After deriving the macroscopic general advection–diffusion equation through the asymptotic homogenization technique, we provide an example application of our model to describe the diffusion of water molecules within packed erythrocytes. This process is characterized by diffusion coefficients with distinct time scales, as observed in prior studies [37, 61]. Diffusion barriers, such as permeable membranes, disrupt the linear relationship between the mean squared displacement and elapsed time [37, 61]. This deviation from standard diffusion laws enables the extraction of detailed information about the underlying biological system. Unlike a constant diffusion coefficient, the time-dependent diffusion coefficient $D(t)$ encapsulates the complexities of molecular motion, shaped by barriers and system geometry. Experimental techniques such as pulsed-field gradient nuclear magnetic resonance (PFG-NMR) [37, 42, 61] and integrative optical imaging [66] measure $D(t)$ to track water dynamics or macromolecular diffusion in tissues. These results can be utilized to improve the understanding of the diffusion process in complex biological tissues, such as the brain and tumor environments [29, 31, 52], or in pathologies like cerebral ischemia, where reduced extracellular space and increased tissue tortuosity lead to lower diffusion coefficients [41, 66].

The duration of diffusion measurement plays a critical role in determining the structural details that influence imaging contrast. Short measurement times capture finer tissue details, whereas longer times reveal larger-scale structural variations [11]. Temporal scales are pivotal in understanding diffusion behavior in biological tissues, as they reflect the transition from free fluid-like behavior at short times to interactions with cellular structures at longer times. This dual time-scale approach could be very important for characterizing tissue dynamics and pathological changes in complex biological systems.

In Sect. 2, we present the foundational equations of our problem. Section 3 introduces the multiscale formulation and applies the asymptotic homogenization technique to derive the corresponding macroscopic equation. In Sect. 4, we compare the solutions of our two time-scale model in the particular case of diffusion (with zero velocities and forces) with those of the steady-state case, highlighting the effects of incorporating dual time scales into the diffusion equation. Finally, in Sect. 5, we show an example of application of our model to describe the diffusion of water molecules within packed erythrocytes using experimental data from [37, 42, 61].

## The advection–diffusion problem

Consider a physical domain $\Omega$ divided into two subregions, each of which is occupied by a phase $\Omega _\alpha$ and $\Omega _\beta$ such that $\Omega =\Omega _\alpha \cup \Omega _\beta$ and $\Omega _\alpha \cap \Omega _\beta =\varnothing$; let $\Gamma =\partial \Omega _\alpha \cap \partial \Omega _\beta$ denote the surface interface between them. The classical conservation law reads

1 $$\begin{aligned} \dfrac{\partial c_\gamma (\tilde{\varvec{x}},\tilde{t})}{\partial \tilde{t}}+\nabla _{\tilde{\varvec{x}}}\cdot \varvec{j}_\gamma (\tilde{\varvec{x}},\tilde{t})=0,\quad \gamma =\alpha ,\beta \end{aligned}$$

with

2 $$\begin{aligned} \varvec{j}_\gamma (\tilde{\varvec{x}},\tilde{t})=-D_{\gamma }(\tilde{\varvec{x}},\tilde{t})\nabla _{\tilde{\varvec{x}}}c_\gamma (\tilde{\varvec{x}},\tilde{t}) +c_\gamma (\tilde{\varvec{x}},\tilde{t})\varvec{u}_\gamma (\tilde{\varvec{x}},\tilde{t}) - \varvec{f}_\gamma (\tilde{\varvec{x}},\tilde{t}), \end{aligned}$$

where $\varvec{u}_\gamma$ is a given velocity and $\varvec{f}_\gamma$ is an additional vector contribution representing a drift driving force whose physical meaning and dimensions are consistent with those of a concentration flux, cf. eq (2). Equation (2) comes from the classical Fick’s law, modified by an advection term and an external force that may interact with the diffusion problem. We can then state our governing equations as follows:

3 $$\begin{aligned} {\left\{ \begin{array}{ll} \dfrac{\partial c_\gamma (\tilde{\varvec{x}},\tilde{t})}{\partial \tilde{t}}+\nabla _{\tilde{\varvec{x}}} \cdot \left( \varvec{u}_\gamma (\tilde{\varvec{x}},\tilde{t})c_\gamma (\tilde{\varvec{x}},\tilde{t})\right) +\nabla _{\tilde{\varvec{x}}}\cdot \left( -D_{\gamma }(\tilde{\varvec{x}},\tilde{t})\nabla _{\tilde{\varvec{x}}}c_\gamma (\tilde{\varvec{x}},\tilde{t})\right) =\nabla _{\tilde{\varvec{x}}} \cdot \varvec{f}_\gamma (\tilde{\varvec{x}},\tilde{t}) \   & \Omega _\gamma ,\\[2ex] \left( -D_\alpha (\tilde{\varvec{x}},\tilde{t})\nabla _{\tilde{\varvec{x}}}c_\alpha (\tilde{\varvec{x}},\tilde{t})+\varvec{u}_\alpha (\tilde{\varvec{x}},\tilde{t}) c_\alpha (\tilde{\varvec{x}},\tilde{t})-\varvec{f}_\alpha (\tilde{\varvec{x}},\tilde{t})\right) \cdot \varvec{n}\\ = \left( -D_\beta (\tilde{\varvec{x}},\tilde{t})\nabla _{\tilde{\varvec{x}}}c_\beta (\tilde{\varvec{x}},\tilde{t})+\varvec{u}_\beta (\tilde{\varvec{x}},\tilde{t}) c_\beta (\tilde{\varvec{x}},\tilde{t})-\varvec{f}_\beta (\tilde{\varvec{x}},\tilde{t})\right) \cdot \varvec{n}=P(c_\alpha (\tilde{\varvec{x}},\tilde{t})-c_\beta (\tilde{\varvec{x}},\tilde{t})) \ & \Gamma , \end{array}\right. } \end{aligned}$$

subject to appropriate initial and boundary conditions, where $\gamma =\alpha , \beta$, $\tilde{\varvec{x}}$ is the space variable and $\tilde{t}$ is the time variable, $c_\gamma (\tilde{\varvec{x}},\tilde{t})$ is the concentration of solutes in the phase $\Omega _\gamma$, $D_{\gamma }(\tilde{\varvec{x}},\tilde{t})$ is the diffusion coefficient of the phase $\Omega _\gamma$, *P* is the diffusive permeability of the interface, $\varvec{n}$ is the *outer normal* related to $\Omega _\beta$. The diffusive membrane permeability *P* could in principle be spatially varying (e.g., in case of membrane heterogeneities), but is herein considered constant for the sake of simplicity. The first equations are the well-known advection–diffusion equations. The second equation is a particular case of the Kedem-Katchalsky equation [19, 52, 71] that describes the solutes concentration exchange between two phases separated by a membrane with no fluid flux; hence, with these conditions, the concentration flux is proportional to the concentration difference between the two phases.

We non-dimensionalize the equations of system (3) introducing the quantities

4 $$\begin{aligned} \tilde{\varvec{x}}=L \tilde{\varvec{x}}', \quad c_\gamma =C_r c'_\gamma , \quad \tilde{t}=t_c \tilde{t}', \quad D_\gamma =D_c D'_\gamma , \quad \varvec{u}_\gamma =U \varvec{u}'_\gamma \quad \varvec{f}_\gamma =F_c \varvec{f}'_\gamma ; \end{aligned}$$

substituting into system (3), we obtain the following non-dimensional equations (dropping the primes)

5 $$\begin{aligned} {\left\{ \begin{array}{ll} P_l\dfrac{\partial c_\gamma (\tilde{\varvec{x}},\tilde{t})}{\partial \tilde{t}}+A_l\nabla _{\tilde{\varvec{x}}} \cdot \left( \varvec{u}_\gamma (\tilde{\varvec{x}},\tilde{t})c_\gamma (\tilde{\varvec{x}},\tilde{t})\right) +\nabla _{\tilde{\varvec{x}}}\cdot \left( -D_{\gamma }(\tilde{\varvec{x}},\tilde{t})\nabla _{\tilde{\varvec{x}}}c_\gamma (\tilde{\varvec{x}},\tilde{t})\right) =F_l \nabla _{\tilde{\varvec{x}}} \cdot \varvec{f}_\gamma (\tilde{\varvec{x}},\tilde{t}) \   & \Omega _\gamma ,\\[2ex] \left( -D_\alpha (\tilde{\varvec{x}},\tilde{t})\nabla _{\tilde{\varvec{x}}}c_\alpha (\tilde{\varvec{x}},\tilde{t})+\varvec{u}_\alpha (\tilde{\varvec{x}},\tilde{t}) c_\alpha (\tilde{\varvec{x}},\tilde{t})-\varvec{f}_\alpha (\tilde{\varvec{x}},\tilde{t})\right) \cdot \varvec{n}\\ = \left( -D_\beta (\tilde{\varvec{x}},\tilde{t})\nabla _{\tilde{\varvec{x}}}c_\beta (\tilde{\varvec{x}},\tilde{t})+\varvec{u}_\beta (\tilde{\varvec{x}},\tilde{t}) c_\beta (\tilde{\varvec{x}},\tilde{t})-\varvec{f}_\beta (\tilde{\varvec{x}},\tilde{t})\right) \cdot \varvec{n}=\tilde{P}(c_\alpha (\tilde{\varvec{x}},\tilde{t})-c_\beta (\tilde{\varvec{x}},\tilde{t})) \   & \Gamma ,\\ \end{array}\right. } \end{aligned}$$

where

6 $$\begin{aligned} P_l=\dfrac{L^2}{t_c D_c}, \quad \tilde{P}=\dfrac{L P}{D_c}, \quad A_l=\dfrac{UL}{D_c}=\dfrac{1}{\textrm{Pe}}, \quad F_l=\dfrac{F_c L}{C_r D_c}. \end{aligned}$$

We notice that $A_l$ is the inverse of the Peclét number Pe.

## Multiscale formulation

In this section, we employ the asymptotic homogenization technique [8, 23, 35] to obtain a multiscale formulation of the system (5) and derive a macroscale model of the latter. We assume there are two characteristic spatial scales: the macroscale $\varvec{x}$ with a characteristic length *L*, and the microscale $\varvec{y}$ with a characteristic length *d*. We postulate that there is a clear separation between these spatial scales, which means

7 $$\begin{aligned} \epsilon =\dfrac{d}{L}\ll 1, \end{aligned}$$

and they have to be considered independent in a formal way: a generic two-scale function $\phi$ can be described by two distinct variables $\phi (\tilde{\varvec{x}})=\phi (\varvec{x},\varvec{y})$. If we non-dimensionalize the two variables $\varvec{x}=L\varvec{x}'$ and $\varvec{y}=d\varvec{y}'$, we have the following classical relationship

8 $$\begin{aligned} \varvec{y}'=\dfrac{\varvec{x}'}{\epsilon }; \end{aligned}$$

where $\varvec{y}'$ is the spatial coordinate that indicates the rapidly-varying variable, while $\varvec{x}'$ indicates the slowly-varying one.

Connected to these two spatial variables there are two different time scales: one related to the characteristic time to cover the length *d* and the other needed to cover the length *L* [9, 43, 47, 48]. A generic two-scale function $\phi$ in time can be written as $\phi (\tilde{\varvec{x}},\tilde{t})=\phi (\varvec{x},\varvec{y},t,\tau )$. If we non-dimensionalize the two time variables with respect to the diffusion characteristic time $\dfrac{d^2}{D_c}$ at the microscale and $\dfrac{L^2}{D_c}$ at the macroscale [8, 43], we have (in non-dimensional form)

9 $$\begin{aligned} \tilde{t}=\dfrac{d^2}{D_c}\tau ', \quad \tilde{t}=\dfrac{L^2}{D_c}t'. \end{aligned}$$

Moreover, we consider as characteristic velocity *U* the one related to the diffusion time scale at the macroscale, such that

10 $$\begin{aligned} U=\dfrac{D_c}{L}, \end{aligned}$$

which implies

11 $$\begin{aligned} A_l = 1. \end{aligned}$$

Hence from Equation (9), we have the relationship

12 $$\begin{aligned} \tau '=\dfrac{t'}{\epsilon ^2}. \end{aligned}$$

From now on, we omit the primes $(')$ to indicate the non-dimensional quantities.

### Remark 1

Due to the fact that we have a multiscale problem with two different spatial scales, we have also two different time scales $t_c$ connected to these two spatial variables: the fast-varying one related to the characteristic diffusion time needed to cover the microscopic length *d* ($t_c^{(1)}=\dfrac{d^2}{D_c}$) and the slow-varying one related to the characteristic diffusion time needed to cover the macroscopic length *L* ($t_c^{(2)}=\dfrac{L^2}{D_c}$) [9, 43, 47, 48]. For this reason, it naturally follows that we have two time scales.

By the relationships above, we assume that $c_\gamma$ and $D_\gamma$ depend on $\varvec{x}, \varvec{y}, t, \tau$ and, due to Eqs. (8) and (12), the differential operators transform in the following way:

13 $$\begin{aligned} \nabla _{\tilde{\varvec{x}}}\rightarrow \nabla _{\varvec{x}}+\dfrac{1}{\epsilon }\nabla _{\varvec{y}},\quad \dfrac{\partial }{\partial \tilde{t}} \rightarrow \dfrac{\partial }{\partial t}+\dfrac{1}{\epsilon ^2}\dfrac{\partial }{\partial \tau }. \end{aligned}$$

### Remark 2

We describe a time-dependent diffusion process (also known as *anomalous diffusion*), by incorporating a sharp time-scale separation of the type $D_\gamma (\varvec{x},\varvec{y},t,\tau )$. Indeed, if we do not account for the microscale $\tau$-dependency explicitly in $D_\gamma$, it can be shown that the time dependency with respect to the microscale does not play a role in the final macroscale equations due to the relationship (12). This is because, at the microscale, the solution is always infinitely distant from the initial condition as $\epsilon \rightarrow 0$, as shown by [9, 43, 47, 48]. This fast and slow time-dependency of the diffusion coefficient generates a new mathematical model that takes into account two different time scales, which can appear in the context of several physical and biological applications, see, e.g., [10, 13, 15, 20, 31, 39, 45, 51, 67, 70, 72, 73].

Regarding the geometry of the multiscale problem, we assume *local periodicity*, which means that $c_\gamma$, $\varvec{f}_\gamma$, $\varvec{u}_\gamma$, and $D_\gamma$ are $\varvec{y}$-periodic, *i.e.,* we can focus on a single portion of the microscale domain. Moreover, we assume *macroscopic uniformity*, which implies ignoring geometric variations within the cell structure and inclusions concerning the coarse-scale variable $\varvec{x}$, i.e., the microstructure is unique. From the macroscopic uniformity it follows that

14 $$\begin{aligned} \nabla _{\varvec{x}}\cdot \int \limits _{\Omega _\gamma }(\bullet )\textrm{d}\varvec{y}= \int \limits _{\Omega _\gamma }\nabla _{\varvec{x}}\cdot (\bullet )\textrm{d}\varvec{y}. \end{aligned}$$

Both these assumptions were made to simplify the calculations, but they are not essential from a formal standpoint and can be removed or replaced with less restrictive hypotheses (see [12, 52]). Moreover, we assume that $c_\gamma (\varvec{x},\varvec{y},t,\tau )$, $\varvec{f}_\gamma (\varvec{x},\varvec{y},t,\tau )$, $\varvec{u}_\gamma (\varvec{x},\varvec{y},t,\tau )$ and $D_\gamma (\varvec{x},\varvec{y},t,\tau )$ are *locally bounded* in $\tau$, that is,

15 $$\begin{aligned}&\lim _{\tau \rightarrow \infty } |c_\gamma (\varvec{x},\varvec{y},t,\tau ) |< +\infty , \quad \lim _{\tau \rightarrow \infty } |D_\gamma (\varvec{x},\varvec{y},t,\tau ) |< +\infty , \nonumber \\&\lim _{\tau \rightarrow \infty } |\varvec{f}_\gamma (\varvec{x},\varvec{y},t,\tau ) |< +\infty , \quad \lim _{\tau \rightarrow \infty } |\varvec{u}_\gamma (\varvec{x},\varvec{y},t,\tau ) |< +\infty , \quad \forall \varvec{x}, \forall \varvec{y}, \forall t. \end{aligned}$$

We employ a power series expansion in $\epsilon$ for the variables of our problem as follows:

16 $$\begin{aligned} c_\gamma (\varvec{x},\varvec{y},t,\tau )\equiv c_\gamma ^\epsilon (\varvec{x},\varvec{y},t,\tau )=\sum _{l=0}^{\infty }c_\gamma ^{(l)}(\varvec{x},\varvec{y},t,\tau )\epsilon ^l \qquad \gamma =\alpha , \beta . \end{aligned}$$

Moreover, we suppose that the forces $\varvec{f}_\gamma$ and the advection velocities $\varvec{u}_\gamma$ are also multiscale in nature, which means that we can employ a power series expansion, *i.e.,*

17 $$\begin{aligned}  &   \varvec{f}_\gamma (\varvec{x},\varvec{y},t,\tau )\equiv \varvec{f}_\gamma ^\epsilon (\varvec{x},\varvec{y},t,\tau )=\sum _{l=0}^{\infty }\varvec{f}_\gamma ^{(l)}(\varvec{x},\varvec{y},t,\tau )\epsilon ^l \qquad \gamma =\alpha , \beta , \end{aligned}$$

18 $$\begin{aligned}  &   \varvec{u}_\gamma (\varvec{x},\varvec{y},t,\tau )\equiv \varvec{u}_\gamma ^\epsilon (\varvec{x},\varvec{y},t,\tau )=\sum _{l=0}^{\infty }\varvec{u}_\gamma ^{(l)}(\varvec{x},\varvec{y},t,\tau )\epsilon ^l \qquad \gamma =\alpha , \beta . \end{aligned}$$

Taking a diffusive characteristic time $t_c=L^2/D_c$, we have from (6) that $P_l$ becomes

19 $$\begin{aligned} P_l= 1. \end{aligned}$$

We also assume that the characteristic force $F_c$ is comparable with the characteristic flux given by diffusion, and for ease of presentation we then set

20 $$\begin{aligned} F_c = \dfrac{C_r D_c}{L}, \end{aligned}$$

which leads to

21 $$\begin{aligned} F_l= 1. \end{aligned}$$

### Remark 3

While we have chosen to set the parameters $P_l=\dfrac{L^2}{t_c D_c}, \quad A_l=\dfrac{UL}{D_c}=\dfrac{1}{Pe}, \quad F_l=\dfrac{F_c L}{C_r D_c}$ to unity for the sake of ease of presentation, the structure of our results remains unaltered as long as those parameters are all of *O*(1), i.e., they do not feature any particular scaling with respect to the smallness parameter $\epsilon$.

According to the Kedem-Katchalsky equations [19, 71], the solute flux $J_s$ across the interface between the two phases without fluid flux can be expressed as

$$\begin{aligned} J_s = \tilde{P} \bar{S} \left( c_\alpha (\varvec{x},\varvec{y},t,\tau ) - c_\beta (\varvec{x},\varvec{y},t,\tau ) \right) , \end{aligned}$$

where $\bar{S}$ represents the total exchange surface density. Since the parameter $d$ characterizes the typical length scale in the microscopic domain, we obtain the proportionality relation

$$\begin{aligned} \bar{S} \propto \frac{L}{d} = \frac{1}{\epsilon }. \end{aligned}$$

Physiologically, the flux measured over a given tissue region is expected to remain finite. Consequently, to ensure a well-defined flux, the interface condition must be scaled by $\epsilon$, as discussed in [52]. Hence we have the following scaling

22 $$\begin{aligned} \tilde{P}=\epsilon \bar{P}, \quad \bar{P}=\dfrac{L \tilde{P}}{d}=\dfrac{L^2 P}{d D_c}. \end{aligned}$$

Substituting (13), (16), and (19) together with the assumptions above, the system (5) becomes

23 $$\begin{aligned}&\epsilon ^2 \dfrac{\partial c^\epsilon _\gamma }{\partial t}(\varvec{x},\varvec{y},t,\tau )+\dfrac{\partial c_\gamma ^\epsilon }{\partial \tau }(\varvec{x},\varvec{y},t,\tau )-\epsilon ^2 \nabla _{\varvec{x}}\cdot \left( D_\gamma (\varvec{x},\varvec{y},t,\tau )\nabla _{\varvec{x}}c^\epsilon _\gamma (\varvec{x},\varvec{y},t,\tau )\right) \nonumber \\&+\epsilon ^2\nabla _{\varvec{x}}\cdot \left( \varvec{u}^\epsilon _\gamma (\varvec{x},\varvec{y},t,\tau )c_\gamma ^\epsilon (\varvec{x},\varvec{y},t,\tau ) \right) +\epsilon \nabla _{\varvec{y}}\cdot \left( \varvec{u}^\epsilon _\gamma (\varvec{x},\varvec{y},t,\tau )c_\gamma ^\epsilon (\varvec{x},\varvec{y},t,\tau ) \right) \nonumber \\  &-\epsilon \nabla _{\varvec{x}} \cdot (D_\gamma (\varvec{x},\varvec{y},t,\tau )\nabla _{\varvec{y}}c^\epsilon _\gamma (\varvec{x},\varvec{y},t,\tau ))-\epsilon \nabla _{\varvec{y}} \cdot (D_\gamma (\varvec{x},\varvec{y},t,\tau )\nabla _{\varvec{x}}c^\epsilon _\gamma (\varvec{x},\varvec{y},t,\tau ))\nonumber \\&-\nabla _{\varvec{y}} \cdot (D_\gamma (\varvec{x},\varvec{y},t,\tau )\nabla _{\varvec{y}}c^\epsilon _\gamma (\varvec{x},\varvec{y},t,\tau ))=\epsilon \nabla _{\varvec{y}} \cdot \varvec{f}^\epsilon _\gamma (\varvec{x},\varvec{y},t,\tau )+\epsilon ^2 \nabla _{\varvec{x}} \cdot \varvec{f}^\epsilon _\gamma (\varvec{x},\varvec{y},t,\tau ) \quad \text {in} \ \Omega _\gamma , \end{aligned}$$

24 $$\begin{aligned}&\left[ -D_\alpha (\varvec{x},\varvec{y},t,\tau )(\epsilon \nabla _{\varvec{x}}c^\epsilon _\alpha (\varvec{x},\varvec{y},t,\tau )+\nabla _{\varvec{y}}c^\epsilon _\alpha (\varvec{x},\varvec{y},t,\tau ))+\epsilon \varvec{u}^\epsilon _\alpha (\tilde{\varvec{x}},\tilde{t}) c^\epsilon _\alpha (\tilde{\varvec{x}},\tilde{t})-\epsilon \varvec{f}^\epsilon _\alpha (\tilde{\varvec{x}},\tilde{t})\right] \cdot \varvec{n}\nonumber \\  &\quad =\left[ -D_\beta (\varvec{x},\varvec{y},t,\tau )(\epsilon \nabla _{\varvec{x}}c^\epsilon _\beta (\varvec{x},\varvec{y},t,\tau )+\nabla _{\varvec{y}}c^\epsilon _\beta (\varvec{x},\varvec{y},t,\tau ))+\epsilon \varvec{u}^\epsilon _\beta (\tilde{\varvec{x}},\tilde{t}) c^\epsilon _\beta (\tilde{\varvec{x}},\tilde{t})-\epsilon \varvec{f}^\epsilon _\beta (\tilde{\varvec{x}},\tilde{t})\right] \cdot \varvec{n}\nonumber \\  &\quad =\epsilon ^2 \bar{P}(c^\epsilon _\alpha (\varvec{x},\varvec{y},t,\tau )-c^\epsilon _\beta (\varvec{x},\varvec{y},t,\tau )) \quad \text{ on } \ \Gamma . \end{aligned}$$

### Coefficient of order $\epsilon ^0$

If we collect the terms of order $\epsilon ^0$ from Eqs. (23) and (24), we obtain

25 $$\begin{aligned}  &   \dfrac{\partial c_\gamma ^{(0)}}{\partial \tau }(\varvec{x},\varvec{y},t,\tau )-\nabla _{\varvec{y}} \cdot \left( D_\gamma (\varvec{x},\varvec{y},t,\tau )\nabla _{\varvec{y}}c^{(0)}_\gamma (\varvec{x},\varvec{y},t,\tau )\right) =0 \quad \text {in} \ \Omega _\gamma , \end{aligned}$$

26 $$\begin{aligned}  &   \left( D_\alpha (\varvec{x},\varvec{y},t,\tau )\nabla _{\varvec{y}}c^{(0)}_\alpha (\varvec{x},\varvec{y},t,\tau )\right) \cdot \varvec{n}= \left( D_\beta (\varvec{x},\varvec{y},t,\tau )\nabla _{\varvec{y}}c^{(0)}_\beta (\varvec{x},\varvec{y},t,\tau )\right) \cdot \varvec{n}=0 \quad \text {on} \ \Gamma . \end{aligned}$$

Due to the uniqueness of the solution of problem (25), we have (for $\gamma =\alpha , \beta$)

27 $$\begin{aligned} c^{(0)}_\gamma (\varvec{x},\varvec{y},t,\tau ) \equiv c^{(0)}_\gamma (\varvec{x},t). \end{aligned}$$

### Coefficient of order $\epsilon ^1$

If we collect the terms of order $\epsilon ^1$ from Eqs. (23) and (24), we obtain

28 $$\begin{aligned}&\dfrac{\partial c^{(1)}_\gamma }{\partial \tau }(\varvec{x},\varvec{y},t,\tau )+\nabla _{\varvec{y}} \cdot \left( \varvec{u}^{(0)}_\gamma (\varvec{x},\varvec{y},t,\tau )c^{(0)}_\gamma (\varvec{x},t)\right) -\nabla _{\varvec{x}} \cdot \left( D_\gamma (\varvec{x},\varvec{y},t,\tau )\nabla _{\varvec{y}}c^{(0)}_\gamma (\varvec{x},t)\right) \nonumber \\  &-\nabla _{\varvec{y}} \cdot \left( D_\gamma (\varvec{x},\varvec{y},t,\tau )\nabla _{\varvec{x}}c^{(0)}_\gamma (\varvec{x},t)\right) -\nabla _{\varvec{y}} \cdot \left( D_\gamma (\varvec{x},\varvec{y},t,\tau )\nabla _{\varvec{y}}c^{(1)}_\gamma (\varvec{x},\varvec{y},t,\tau )\right) = \nabla _{\varvec{y}} \cdot \varvec{f}^{(0)}_\gamma (\varvec{x},\varvec{y},t,\tau ) \quad \text {in} \ \Omega _\gamma , \end{aligned}$$

which becomes

29 $$\begin{aligned} \dfrac{\partial c^{(1)}_\gamma }{\partial \tau }(\varvec{x},\varvec{y},t,\tau )&+\nabla _{\varvec{y}} \cdot \left( \varvec{u}^{(0)}_\gamma (\varvec{x},\varvec{y},t,\tau )c^{(0)}_\gamma (\varvec{x},t)\right) -\nabla _{\varvec{y}}D_\gamma (\varvec{x},\varvec{y},t,\tau ) \nabla _{\varvec{x}}c^{(0)}_\gamma (\varvec{x},t)\nonumber \\  &-\nabla _{\varvec{y}} \cdot \left( D_\gamma (\varvec{x},\varvec{y},t,\tau )\nabla _{\varvec{y}}c^{(1)}_\gamma (\varvec{x},\varvec{y},t,\tau )\right) = \nabla _{\varvec{y}} \cdot \varvec{f}^{(0)}_\gamma (\varvec{x},\varvec{y},t,\tau ) \quad \text{ in } \ \Omega _\gamma ,\end{aligned}$$

and

30 $$\begin{aligned}&\left( -D_\alpha (\varvec{x},\varvec{y},t,\tau )\left( \nabla _{\varvec{x}}c^{(0)}_\alpha (\varvec{x},t)+\nabla _{\varvec{y}}c^{(1)}_\alpha (\varvec{x},\varvec{y},t,\tau ) \right) +\varvec{u}^{(0)}_\alpha (\varvec{x},\varvec{y},t,\tau ) c^{(0)}_\alpha (\varvec{x},t)-\varvec{f}^{(0)}_\alpha (\varvec{x},\varvec{y},t,\tau )\right) \cdot \varvec{n}\nonumber \\  &\quad =\left( -D_\beta (\varvec{x},\varvec{y},t,\tau )\left( \nabla _{\varvec{x}}c^{(0)}_\beta (\varvec{x},t)+\nabla _{\varvec{y}}c^{(1)}_\beta (\varvec{x},\varvec{y},t,\tau ) \right) +\varvec{u}^{(0)}_\beta (\varvec{x},\varvec{y},t,\tau ) c^{(0)}_\beta (\varvec{x},t)-\varvec{f}^{(0)}_\beta (\varvec{x},\varvec{y},t,\tau )\right) \nonumber \\&\quad \cdot \varvec{n}=0 \quad \text{ on } \ \Gamma . \end{aligned}$$

We notice that the problem is linear and that $\nabla _{\varvec{x}}c^{(0)}$ is constant in $\varvec{y}$ and $\tau$, hence we formulate the following solution ansatz:

31 $$\begin{aligned} c_\gamma ^{(1)}(\varvec{x},\varvec{y},t,\tau )=\varvec{a}_\gamma (\varvec{x},\varvec{y},t,\tau )\cdot \nabla _{\varvec{x}} c^{(0)}_\gamma (\varvec{x},t)+\tilde{a}_\gamma (\varvec{x},\varvec{y},t,\tau )+\bar{d}_\gamma (\varvec{x},\varvec{y},t,\tau ) c_\gamma ^{(0)}(\varvec{x},t). \end{aligned}$$

The ansatz (31) solves the problem (29)–(30) (it is a solution up to a $\varvec{y}$-constant function), provided that the auxiliary vector and scalar fields $\varvec{a}_\gamma$, $\tilde{a}_\gamma$, $\bar{d}_\gamma$ solve the following cell problems

32 $$\begin{aligned}  &   {\left\{ \begin{array}{ll} \dfrac{\partial \varvec{a}_\gamma }{\partial \tau }(\varvec{x},\varvec{y},t,\tau )-\nabla _{\varvec{y}} D_\gamma (\varvec{x},\varvec{y},t,\tau )-\nabla _{\varvec{y}} \cdot \left( D_\gamma (\varvec{x},\varvec{y},t,\tau )\nabla _{\varvec{y}}\varvec{a}_\gamma (\varvec{x},\varvec{y},t,\tau )\right) =0 \quad & \text {in} \ \Omega _\gamma ,\\ \left[ D_\gamma (\varvec{x},\varvec{y},t,\tau )\left( \nabla _{\varvec{y}} \varvec{a}_\gamma (\varvec{x},\varvec{y},t,\tau )+\mathbb {I}\right) \right] \varvec{n}=\varvec{0} \quad & \text {on} \ \Gamma , \end{array}\right. } \end{aligned}$$

33 $$\begin{aligned}  &   {\left\{ \begin{array}{ll} \dfrac{\partial \tilde{a}_\gamma }{\partial \tau }(\varvec{x},\varvec{y},t,\tau )-\nabla _{\varvec{y}} \cdot \left( D_\gamma (\varvec{x},\varvec{y},t,\tau )\nabla _{\varvec{y}}\tilde{a}_\gamma (\varvec{x},\varvec{y},t,\tau )\right) = \nabla _{\varvec{y}}\cdot \varvec{f}^{(0)}_\gamma (\varvec{x},\varvec{y},t,\tau ) \quad & \text {in} \ \Omega _\gamma ,\\ \left( D_\gamma (\varvec{x},\varvec{y},t,\tau )\nabla _{\varvec{y}} \tilde{a}_\gamma (\varvec{x},\varvec{y},t,\tau )\right) \cdot \varvec{n}=-\varvec{f}^{(0)}_\gamma (\varvec{x},\varvec{y},t,\tau )\cdot \varvec{n}\quad & \text {on} \ \Gamma , \end{array}\right. } \end{aligned}$$

34 $$\begin{aligned}  &   {\left\{ \begin{array}{ll} \dfrac{\partial \bar{d}_\gamma }{\partial \tau }(\varvec{x},\varvec{y},t,\tau )+\nabla _{\varvec{y}} \cdot \varvec{u}^{(0)}_\gamma (\varvec{x},\varvec{y},t,\tau )-\nabla _{\varvec{y}} \cdot \left( D_\gamma (\varvec{x},\varvec{y},t,\tau )\bar{d}_\gamma (\varvec{x},\varvec{y},t,\tau )\right) = 0 \quad &  \text{ in } \ \Omega _\gamma ,\\ \left( D_\gamma (\varvec{x},\varvec{y},t,\tau )\nabla _{\varvec{y}} \bar{d}_\gamma (\varvec{x},\varvec{y},t,\tau )\right) \cdot \varvec{n}=\varvec{u}_\gamma ^{(0)}(\varvec{x},\varvec{y},t,\tau )\cdot \varvec{n} \quad &  \text{ on } \ \Gamma , \end{array}\right. } \end{aligned}$$

where $\mathbb {I}$ is the second order identity tensor, and we impose $<\varvec{a}_\gamma >_{\Omega _\gamma }=\varvec{0}$, $<\tilde{a}_\gamma >_{\Omega _\gamma }=0$, and $<\bar{d}_\gamma >_{\Omega _\gamma }=0$ to ensure the uniqueness of the solution, where $<\cdot >_{\Omega _\gamma }$ is the average operator defined by

35 $$\begin{aligned} <\cdot >_{\Omega _\gamma } = \dfrac{1}{|\Omega _\gamma |}\int \limits _{\Omega _\gamma }(\cdot ) \textrm{d}\varvec{y}. \end{aligned}$$

Moreover, we define an average operator for the microscale time $\tau$ as follows (inspired by [12]):

36 $$\begin{aligned} <h(\varvec{x},\varvec{y},t,\tau )>_\tau = \lim _{\hat{t}\rightarrow +\infty }\dfrac{1}{\hat{t}}\int \limits _{[0,\hat{t}]}h(\varvec{x},\varvec{y},t,\tau ) \textrm{d}\tau , \end{aligned}$$

for a generic function *h*. Equation (36) represents the average value calculated over a long period for a single instance of a function in space, while (35) is the spatial average for a single point in time. Comparisons between averages of this kind can appear when dealing with ergodic systems (where there is an equivalency between those averages), although this is in general not the case here.

#### Remark 4

If we consider the average $<\cdot >_\tau$ of (32), we note that the problem (and the solution) is different from the steady one due to the dependence on $\tau$ of the diffusive coefficient $D_\gamma$. However, if $D_\gamma$ were not depending on $\tau$, the microscale time average $<\cdot >_\tau$ would lead to the classical steady diffusion problem in terms of the averaged auxiliary variable $<\varvec{a}_\gamma (\varvec{x},\varvec{y},t,\tau )>_\tau$.

### Coefficient of order $\epsilon ^2$

Collecting the terms of order $\epsilon ^2$ from Eqs. (23) and (24), we have

37 $$\begin{aligned}&\dfrac{\partial c_\gamma ^{(0)}}{\partial t}(\varvec{x},t){+}\dfrac{\partial c^{(2)}_\gamma }{\partial \tau }(\varvec{x},\varvec{y},t,\tau ){-}\nabla _{\varvec{x}} \cdot \left( D_\gamma (\varvec{x},\varvec{y},t,\tau )\nabla _{\varvec{y}}c^{(1)}_\gamma (\varvec{x},\varvec{y},t,\tau )\right) {-}\nabla _{\varvec{y}} \cdot \left( D_\gamma (\varvec{x},\varvec{y},t,\tau )\nabla _{\varvec{x}}c^{(1)}_\gamma (\varvec{x},\varvec{y},t,\tau )\right) \nonumber \\  &\quad + \nabla _{\varvec{x}}\cdot \left( c_\gamma ^{(0)}(\varvec{x},\varvec{y},t,\tau )\varvec{u}_\gamma ^{(0)}(\varvec{x},\varvec{y},t,\tau )\right) +\nabla _{\varvec{y}}\cdot \left( c_\gamma ^{(1)}(\varvec{x},\varvec{y},t,\tau )\varvec{u}_\gamma ^{(0)}(\varvec{x},\varvec{y},t,\tau )\right) \nonumber \\  &\quad + \nabla _{\varvec{y}}\cdot \left( c_\gamma ^{(0)}(\varvec{x},\varvec{y},t,\tau )\varvec{u}_\gamma ^{(1)}(\varvec{x},\varvec{y},t,\tau )\right) \nonumber \\  &\quad -\nabla _{\varvec{y}} \cdot \left( D_\gamma (\varvec{x},\varvec{y},t,\tau )\nabla _{\varvec{y}}c^{(2)}_\gamma (\varvec{x},\varvec{y},t,\tau )\right) -\nabla _{\varvec{x}}\cdot \left( D_\gamma (\varvec{x},\varvec{y},t,\tau )\nabla _{\varvec{x}}c^{(0)}_\gamma (\varvec{x},t)\right) =\nabla _{\varvec{x}} \cdot \varvec{f}^{(0)}_\gamma (\varvec{x},\varvec{y},t,\tau ) \nonumber \\  &\quad +\nabla _{\varvec{y}} \cdot \varvec{f}^{(1)}_\gamma (\varvec{x},\varvec{y},t,\tau ) \quad \text{ in } \ \Omega _\gamma , \end{aligned}$$

38 $$\begin{aligned}&\big [-D_\alpha (\varvec{x},\varvec{y},t,\tau )\left( \nabla _{\varvec{x}}c^{(1)}_\alpha (\varvec{x},\varvec{y},t,\tau )+\nabla _{\varvec{y}}c^{(2)}_\alpha (\varvec{x},\varvec{y},t,\tau ) \right) \nonumber \\  &\quad +\varvec{u}_\alpha ^{(1)}(\varvec{x},\varvec{y},t,\tau )c_\alpha ^{(0)}(\varvec{x},\varvec{y},t,\tau )+\varvec{u}_\alpha ^{(0)}(\varvec{x},\varvec{y},t,\tau )c_\alpha ^{(1)}(\varvec{x},\varvec{y},t,\tau )-\varvec{f}_\alpha ^{(1)}(\varvec{x},\varvec{y},t,\tau )\big ]\cdot \varvec{n}\nonumber \\  &\quad =\big [-D_\beta (\varvec{x},\varvec{y},t,\tau )\left( \nabla _{\varvec{x}}c^{(1)}_\beta (\varvec{x},\varvec{y},t,\tau )+\nabla _{\varvec{y}}c^{(2)}_\beta (\varvec{x},\varvec{y},t,\tau ) \right) \nonumber \\  &\quad + \varvec{u}_\beta ^{(1)}(\varvec{x},\varvec{y},t,\tau )c_\beta ^{(0)}(\varvec{x},\varvec{y},t,\tau )+\varvec{u}_\beta ^{(0)}(\varvec{x},\varvec{y},t,\tau )c_\beta ^{(1)}(\varvec{x},\varvec{y},t,\tau )-\varvec{f}_\beta ^{(1)}(\varvec{x},\varvec{y},t,\tau )\big ]\cdot \varvec{n}\nonumber \\  &\quad =\bar{P}\left( c_\alpha ^{(0)}(\varvec{x},t)-c_\beta ^{(0)}(\varvec{x},t)\right) \quad \text{ on } \ \Gamma . \end{aligned}$$

### The resulting partial differential equations at the macroscale

Taking the average $<\cdot >_{\Omega _\gamma }$ of Eq. (37), substituting the interface condition (38) and applying the average $<\cdot >_{\tau }$, using the divergence theorem with respect to $\varvec{y}$ and recalling that $\varvec{n}$ is the outer normal to $\Omega _\beta$, by means of the periodicity assumption on the outer boundaries of the cell, Equation (37) leads to the following differential equations (we note that in the following equations we have declared the indices corresponding to the phases $\alpha , \beta$ explicitly rather than utilizing the index $\gamma$):

39 $$\begin{aligned}&\dfrac{\partial c_\alpha ^{(0)}}{\partial t}(\varvec{x},t)-\nabla _{\varvec{x}} \cdot \left( \bar{D}_\alpha (\varvec{x},t)\nabla _{\varvec{x}}c_\alpha ^{(0)}(\varvec{x},t)\right) +S\bar{P}\left( c_\alpha ^{(0)}(\varvec{x},t)-c_{\beta }^{(0)}(\varvec{x},t)\right) \nonumber \\  &\quad +\nabla _{\varvec{x}}\cdot \left( \langle \langle \varvec{u}^{(0)}_\alpha (\varvec{x},\varvec{y},t,\tau )-D_\alpha (\varvec{x},\varvec{y},t,\tau )\nabla _{\varvec{y}}\bar{d}_\alpha (\varvec{x},\varvec{y},t,\tau )\rangle _{\Omega _\alpha }\rangle _{\tau }c_\alpha ^{(0)}(\varvec{x},t)\right) \nonumber \\  &\quad =\nabla _{\varvec{x}} \cdot \langle \langle D_\alpha (\varvec{x},\varvec{y},t,\tau )\nabla _{\varvec{y}} \tilde{a}_\alpha (\varvec{x},\varvec{y},t,\tau )\rangle _{\Omega _\alpha }\rangle _{\tau }+ \langle \langle \nabla _{\varvec{x}}\cdot \varvec{f}^{(0)}_\alpha (\varvec{x},\varvec{y},t,\tau )\rangle _{\Omega _\alpha }\rangle _{\tau }, \end{aligned}$$

40 $$\begin{aligned}&\dfrac{\partial c_\beta ^{(0)}}{\partial t}(\varvec{x},t)-\nabla _{\varvec{x}} \cdot \left( \bar{D}_\beta (\varvec{x},t)\nabla _{\varvec{x}}c_\beta ^{(0)}(\varvec{x},t)\right) +S\bar{P}\left( c_\beta ^{(0)}(\varvec{x},t)-c_{\alpha }^{(0)}(\varvec{x},t)\right) \nonumber \\  &\quad +\nabla _{\varvec{x}}\cdot \left( \langle \langle \varvec{u}^{(0)}_\beta (\varvec{x},\varvec{y},t,\tau )-D_\beta (\varvec{x},\varvec{y},t,\tau )\nabla _{\varvec{y}}\bar{d}_\beta (\varvec{x},\varvec{y},t,\tau )\rangle _{\Omega _\beta }\rangle _{\tau }c_\beta ^{(0)}(\varvec{x},t)\right) \nonumber \\  &\quad =\nabla _{\varvec{x}} \cdot \langle \langle D_\beta (\varvec{x},\varvec{y},t,\tau )\nabla _{\varvec{y}} \tilde{a}_\beta (\varvec{x},\varvec{y},t,\tau )\rangle _{\Omega _\beta }\rangle _{\tau }+ \langle \langle \nabla _{\varvec{x}} \cdot \varvec{f}^{(0)}_\beta (\varvec{x},\varvec{y},t,\tau )\rangle _{\Omega _\beta }\rangle _{\tau }, \end{aligned}$$

where *S* is the non-dimensional surface of the interface between the two phases such that

41 $$\begin{aligned} S=\dfrac{S^\text {tot}d}{|\Omega |}, \end{aligned}$$

where $S^\text {tot}$ is the total area of the interface surface, and $|\Omega |$ is the total macroscopic volume. Moreover, we have

42 $$\begin{aligned}  &   \bar{D}_\alpha (\varvec{x},t)=<<D_\alpha (\varvec{x},\varvec{y},t,\tau )\mathbb {I}>_{\Omega _\alpha }>_\tau +<<D_\alpha (\varvec{x},\varvec{y},t,\tau )\nabla _{\varvec{y}}\varvec{a}_\alpha (\varvec{x},\varvec{y},t,\tau )>_{\Omega _\alpha }>_\tau , \end{aligned}$$

43 $$\begin{aligned}  &   \bar{D}_\beta (\varvec{x},t)=<<D_\beta (\varvec{x},\varvec{y},t,\tau )\mathbb {I}>_{\Omega _\beta }>_\tau +<<D_\beta (\varvec{x},\varvec{y},t,\tau )\nabla _{\varvec{y}}\varvec{a}_\beta (\varvec{x},\varvec{y},t,\tau )>_{\Omega _\beta }>_\tau , \end{aligned}$$

where $\mathbb {I}$ is the second-order identity tensor.

The average operators introduced in Eqs. (39) and (40) commute in the case under investigation. However, they may not in the more general circumstance in which $\Omega _\alpha$ and/or $\Omega _\beta$ evolve in time (assume, for example, that the interface between the two phases moves), as in [53]. Equations (39) and (40) differ from the classical advection–diffusion equations derived using the asymptotic homogenization technique at the macroscale [52, 63] due to several key factors. These include the dependence on both microscale and macroscale time, which arises from the time-dependent diffusion coefficient, as well as the presence of additional multiscale forces and source terms linked to the nonzero divergence of velocity.

The influence of a time-dependent diffusion coefficient has been previously explored in [18, 34], while multiscale forces were first introduced in [56]. In this work, we investigate both the dual time-dependence of the diffusion coefficient and the role of multiscale forces, which lead to the formulation of the cell problems (32) and (33), respectively, along with the inclusion of additional source terms.

Furthermore, our macroscopic model incorporates a novel source term associated with the nonzero divergence of velocity. This term modifies the macroscopic advection velocity by accounting for source and sink effects at the microscale. Its presence necessitates the introduction of a new cell problem, given by (34).

Finally, we observe that the problem (33) is driven by the external forces, and the solution reduces to zero (or a $\textbf{y}$-constant function if a different uniqueness condition is chosen), whenever this is the case, hence the additional contributions due to local variations of the forces related to $\nabla _{\varvec{y}} \tilde{a}_\gamma (\varvec{x},\varvec{y},t,\tau )$ with $\gamma =\alpha ,\, \beta$ also vanish accordingly.

Similarly, if the microscale velocity field satisfies $\nabla _{\varvec{y}} \cdot \varvec{u}^{(0)} = 0$, the solution of (34) also becomes zero, so that the correction to the macroscale velocity related to $\nabla _{\varvec{y}} \bar{d}_\gamma (\varvec{x},\varvec{y},t,\tau )$ with $\gamma =\alpha ,\, \beta$ is no longer present and in this case the macroscale velocity merely coincides with the cell average of the leading order velocity field.

If we substitute the non-dimensionalization (4) and (41) to the problems (39) and (40), we obtain the following macroscopic dimensional problems

44 $$\begin{aligned}&\dfrac{\partial c_\alpha ^{(0)}}{\partial t}(\varvec{x},t)-\nabla _{\varvec{x}} \cdot \left( \bar{D}_\alpha (\varvec{x},t)\nabla _{\varvec{x}}c_\alpha ^{(0)}(\varvec{x},t)\right) +\dfrac{S^\text {tot}P}{|\Omega |}\left( c_\alpha ^{(0)}(\varvec{x},t)-c_{\beta }^{(0)}(\varvec{x},t)\right) \nonumber \\  &\quad +\nabla _{\varvec{x}}\cdot \left( \langle \langle \varvec{u}^{(0)}_\alpha (\varvec{x},\varvec{y},t,\tau )-D_\alpha (\varvec{x},\varvec{y},t,\tau )\nabla _{\varvec{y}}\bar{d}_\alpha (\varvec{x},\varvec{y},t,\tau )\rangle _{\Omega _\alpha }\rangle _{\tau }c_\alpha ^{(0)}(\varvec{x},t)\right) \nonumber \\  &\quad =\nabla _{\varvec{x}} \cdot \langle \langle D_\alpha (\varvec{x},\varvec{y},t,\tau )\nabla _{\varvec{y}} \tilde{c}_\alpha (\varvec{x},\varvec{y},t,\tau )\rangle _{\Omega _\alpha }\rangle _{\tau }+ \langle \langle \nabla _{\varvec{x}} \cdot \varvec{f}^{(0)}_\alpha (\varvec{x},\varvec{y},t,\tau )\rangle _{\Omega _\alpha }\rangle _{\tau }, \end{aligned}$$

45 $$\begin{aligned}&\dfrac{\partial c_\beta ^{(0)}}{\partial t}(\varvec{x},t)-\nabla _{\varvec{x}} \cdot \left( \bar{D}_\beta (\varvec{x},t)\nabla _{\varvec{x}}c_\beta ^{(0)}(\varvec{x},t)\right) +\dfrac{S^\text {tot}P}{|\Omega |}\left( c_\beta ^{(0)}(\varvec{x},t)-c_{\alpha }^{(0)}(\varvec{x},t)\right) \nonumber \\  &\quad +\nabla _{\varvec{x}}\cdot \left( \langle \langle \varvec{u}^{(0)}_\beta (\varvec{x},\varvec{y},t,\tau )-D_\beta (\varvec{x},\varvec{y},t,\tau )\nabla _{\varvec{y}}\bar{d}_\beta (\varvec{x},\varvec{y},t,\tau )\rangle _{\Omega _\beta }\rangle _{\tau }c_\beta ^{(0)}(\varvec{x},t)\right) \nonumber \\  &\quad =\nabla _{\varvec{x}} \cdot \langle \langle D_\beta (\varvec{x},\varvec{y},t,\tau )\nabla _{\varvec{y}} \tilde{c}_\beta (\varvec{x},\varvec{y},t,\tau )\rangle _{\Omega _\beta }\rangle _{\tau }+ \langle \langle \nabla _{\varvec{x}}\cdot \varvec{f}^{(0)}_\beta (\varvec{x},\varvec{y},t,\tau )\rangle _{\Omega _\beta }\rangle _{\tau }, \end{aligned}$$

where $\tilde{c}_\gamma =C_r\tilde{a}_\gamma$. These dimensional problems can be useful in the modeling of real phenomena with data from the literature.

## Numerical test: 2D case

Since the time-dependent component of the diffusion coefficient $D_\gamma (\varvec{x},\varvec{y},t,\tau )$ has not been explicitly investigated numerically, in this section and the next, we present numerical simulations focusing solely on the diffusion equation, i.e., with $\varvec{u}_\gamma = \varvec{0}$ and $\varvec{f}_\gamma = \varvec{0}$. The general case will be addressed in future studies.

We test the anomalous diffusion model to clarify the differences between using a steady diffusion coefficient and a time-dependent diffusion coefficient. We focus on one phase ($\gamma$ is then omitted), and the only boundary conditions for the cell problem (32) are the periodic boundary conditions (given by the local periodicity).

We fix a rectangular microscale geometry $[0,a]\times [0,b]$, and for the steady diffusion coefficient we use the well-known form [23]

46 $$\begin{aligned} D(\varvec{x},\varvec{y},t,\tau )\equiv D(\varvec{y}) \equiv D(y_1,y_2)=\mu \left( 1+\delta \sin \left( \dfrac{y_1}{a\epsilon }\right) \sin \left( \dfrac{y_2}{b\epsilon }\right) \right) . \end{aligned}$$

For comparison with the above steady case, we define these three time-dependent diffusion coefficients

47 $$\begin{aligned} D(\varvec{x},\varvec{y},t,\tau ) \equiv D(\varvec{y},\tau )\equiv &   D(y_1,y_2,\tau )\nonumber \\  = &   \mu \left( 1+\delta \sin \left( \dfrac{y_1}{a\epsilon }\right) \sin \left( \dfrac{y_2}{b\epsilon }\right) \right) +\mu \left( 1+\delta \sin \left( \dfrac{y_1}{a\epsilon }\right) \sin \left( \dfrac{y_2}{b\epsilon }\right) \right) \dfrac{\sin (\pi \tau )}{2}; \end{aligned}$$

48 $$\begin{aligned} D(\varvec{x},\varvec{y},t,\tau ) \equiv D(\varvec{y},\tau ) \equiv D(y_1,y_2,\tau )&=\mu \left( 1+\delta \sin \left( \dfrac{y_1}{a\epsilon }\right) \sin \left( \dfrac{y_2}{b\epsilon }\right) \right) \nonumber \\  &\quad +\mu \left( 1+\delta \sin \left( \dfrac{y_1}{a\epsilon }\right) \sin \left( \dfrac{y_2}{b\epsilon }\right) \right) \left( \sin ^2(\pi \tau )-\dfrac{1}{2}\right) ; \end{aligned}$$

49 $$\begin{aligned} D(\varvec{x},\varvec{y},t,\tau ) \equiv D(\varvec{y},\tau ) \equiv D(y_1,y_2,\tau )&=\mu \left( 1+\delta \sin \left( \dfrac{y_1}{a\epsilon }\right) \sin \left( \dfrac{y_2}{b\epsilon }\right) \right) \nonumber \\  &\quad +\mu \left( 1+\delta \sin \left( \dfrac{y_1}{a\epsilon }\right) \sin \left( \dfrac{y_2}{b\epsilon }\right) \right) \left( (\sin (\pi \tau ))^+-\dfrac{2}{\pi }\right) ; \end{aligned}$$

where $()^+$ is the positive part. In Eqs. (47), (48), and (49) we added a zero-mean term in time with respect to the steady diffusion coefficient (46). We set the parameters as follows: $a=b=1$, $\delta =0.9$, $\mu =1$, $\epsilon =1$. The effective diffusion coefficients $\bar{D}$ in the steady case for only one phase defined in either Equation (42) or (43) is found numerically and it is as follows

50 $$\begin{aligned} \bar{D}=\begin{bmatrix} 0.883536 & \quad 0 \\ 0 & \quad 0.883536 \end{bmatrix}. \end{aligned}$$

We now perform the same numerical test by utilizing the three time dependent diffusion coefficients Eqs. (47)–(49) in place of the steady diffusion coefficient (46). By keeping otherwise the same set of parameters (in particular $a=b$), we then obtain diagonal and spherical (i.e., scalar multiple of the identity) effective diffusivity tensors. Hence, we can then compare the diagonal of the matrix representing the effective diffusivity tensor (50) in the steady case with the one obtained for the time-dependent diffusion coefficients (42)–(43). The diagonal component of the effective diffusion coefficient $\bar{D}$ (42)–(43) in the three time-dependent cases is indeed different and given by: 0.9995, 0.9392 and 0.6336 for the cases (47), (48), (49), respectively.

The above analysis refers to a very simple benchmark exploiting a 2D geometry. In the next section, we will apply the model to a more realistic 3D geometry and by means of a different microscale diffusion coefficient related to a real-world problem of physiological interest.

## Biological application: water diffusion in packed erythrocytes

In this section, we show an example of the application of the model developed in Sects. 2 and 3 with $\varvec{u}_\gamma = \varvec{0}$ and $\varvec{f}_\gamma = \varvec{0}$ to describe water diffusion in packed erythrocytes. This is one of many applications of the proposed model. As pointed out in various papers [37, 41, 42, 61], the diffusion coefficient in packed erythrocytes depends on the fast-varying microscale time and it is useful to measure the surface-to-volume ratio. In [37], the authors found that, for short times, the diffusion coefficient has the form

$$\begin{aligned} D(\tau )=D_0\left( 1-\dfrac{4S\sqrt{D_0 \tau }}{9\sqrt{\pi }V}\right) ,\end{aligned}$$

where *S*/*V* is the surface-to-volume ratio and

$$\begin{aligned} D_0 = {\left\{ \begin{array}{ll} D_\text {int} & \text {inside erythrocytes}\\ D_\text {ext} & \text {outside erythrocytes}. \end{array}\right. } \end{aligned}$$

Moreover, for long times, they established that $\lim _{\tau \rightarrow \infty }D(\tau )=D_\text {eff}$. Later on, in [61] the Padé approximation is employed to describe a diffusion coefficient $D(\tau |t)$ with the above characteristics. In this work, we use the following diffusion coefficient (which is piece-wise constant in space at the microscale as it only varies across the interface between the erythrocytes and the surrounding) as stated in [37, 61] in non-dimensional form, *i.e.,*

51 $$\begin{aligned} D(\varvec{x},\varvec{y},t,\tau )\equiv D(\tau )= \dfrac{D_0}{D_\text {eff}} \left( \dfrac{1+c_1\sqrt{D_0 \tau }+c_2D_0 \tau }{1+\sqrt{D_0\tau }\left( c_1+\dfrac{8}{9\sqrt{\pi }}\dfrac{S}{V}\right) +c_2\alpha _k D_0 \tau }\right) , \end{aligned}$$

where $c_1$ and $c_2$ are parameters of Padé interpolation [61] and we non-dimensionalize the diffusion coefficient given by [61] with respect to $D_\text {eff}$.

Using the data obtained from mice experiment in [42], we have the following parameters: the internal diffusion coefficient $D_\text {int}=9.42 \times 10^{-5}\,\text {cm}^2/\text {s}$, the external diffusion coefficient $D_\text {ext}=1.57 \times 10^{-4}\,\text {cm}^2/\text {s}$. Moreover, we have that, at long times, the diffusion coefficient $D_\text {ext}$ tends to $D^\text {ext}_\text {eff}=7 \times 10^{-5}\,\text {cm}^2/\text {s}$, and $D_\text {int}$ tends to $D^\text {int}_\text {eff}=1 \times 10^{-5}\,\text {cm}^2/\text {s}$, hence by the formula (51), we have $\alpha _k=\dfrac{D_0}{D^\text {int/ext}_\text {eff}}$.

**Fig. 1:**
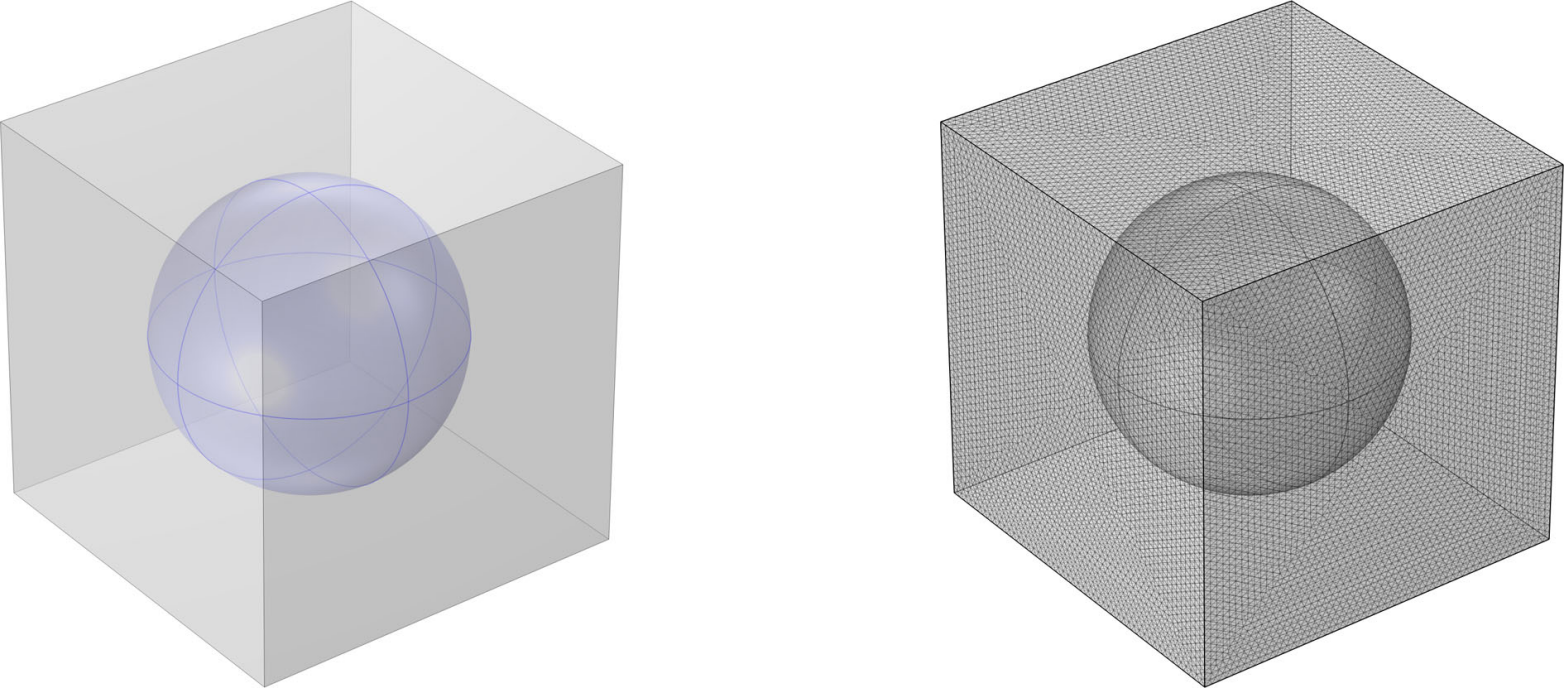
The geometry of the cell related to the cell problem. On the left, the blue sphere represents the erythrocytes geometry, while the gray part represents the extracellular fluid geometry. On the right, we can see the mesh used for the study

We estimate the geometrical parameters using the available data in [42] and supposing a periodic microstructure as shown in Fig. 1. We have, from [42], that the surface-to-volume ratio for the internal and external regions are the following: $(S/V)^\text {int}=1.53\times \,1/ \upmu \text {m}$ and $(S/V)^\text {ext}=0.42\times \,1/ \upmu \text {m}$. Considering a geometry as in Fig. 1 with *l* the length side of the cube and *R* the sphere radius, we have the following relationship

52 $$\begin{aligned} {\left\{ \begin{array}{ll} (S/V)^\text {int}=1.53\times \,1/ \upmu \text {m}=\dfrac{3}{R};\\ (S/V)^\text {ext}=0.42\times \,1/ \upmu \text {m}=\dfrac{4\pi R^2}{l^3-(4/3)\pi R^3}, \end{array}\right. } \end{aligned}$$

we obtain $R\approx 1.96\, \upmu \text {m}$ and $l\approx 3.98\, \upmu \text {m}$. We are working in a non-dimensional case, hence we choose as $d=l$, which means $l'=1$ and $R'=R/d=R/l$.

To find Padé constants $c_1$ and $c_2$, we use a least-square fitting of the curve (51) with the following curve presented in [42]:

53 $$\begin{aligned} D^\text {int/ext}(\tau )=D^\text {int/ext}_\text {eff}+(D^\text {int/ext}_0-D^\text {int/ext}_\text {eff})e^{\left( -4\sqrt{D^\text {int/ext}_0 \tau }/(\sqrt{\pi }\lambda ^\text {int/ext})\right) }, \end{aligned}$$

so that we get $c_1=-0.0191$ and $c_2=0.1452$ for the internal problem, while $c_1=0.1212$ and $c_2=0.0243$ for the external problem. We solve the cell problem (32) to find the macroscopic diffusion coefficients $\bar{D}_\gamma$ defined in (42) and (43) for both phases $\gamma =\alpha$ (the internal phase) and $\gamma =\beta$ (the external phase). We find the following results:

54 $$\begin{aligned} \bar{D}_\alpha =1.0444, \quad \bar{D}_\beta =0.28398. \end{aligned}$$

The surface-to-volume ratio (*S*/*V*) is a critical parameter for understanding the water diffusion coefficient in biological tissues, as it influences the diffusion rate by modulating the interactions at surfaces such as cell membranes [37, 42, 61]. A higher *S*/*V* ratio means increased surface interactions, which restrict water molecule mobility.

**Fig. 2:**
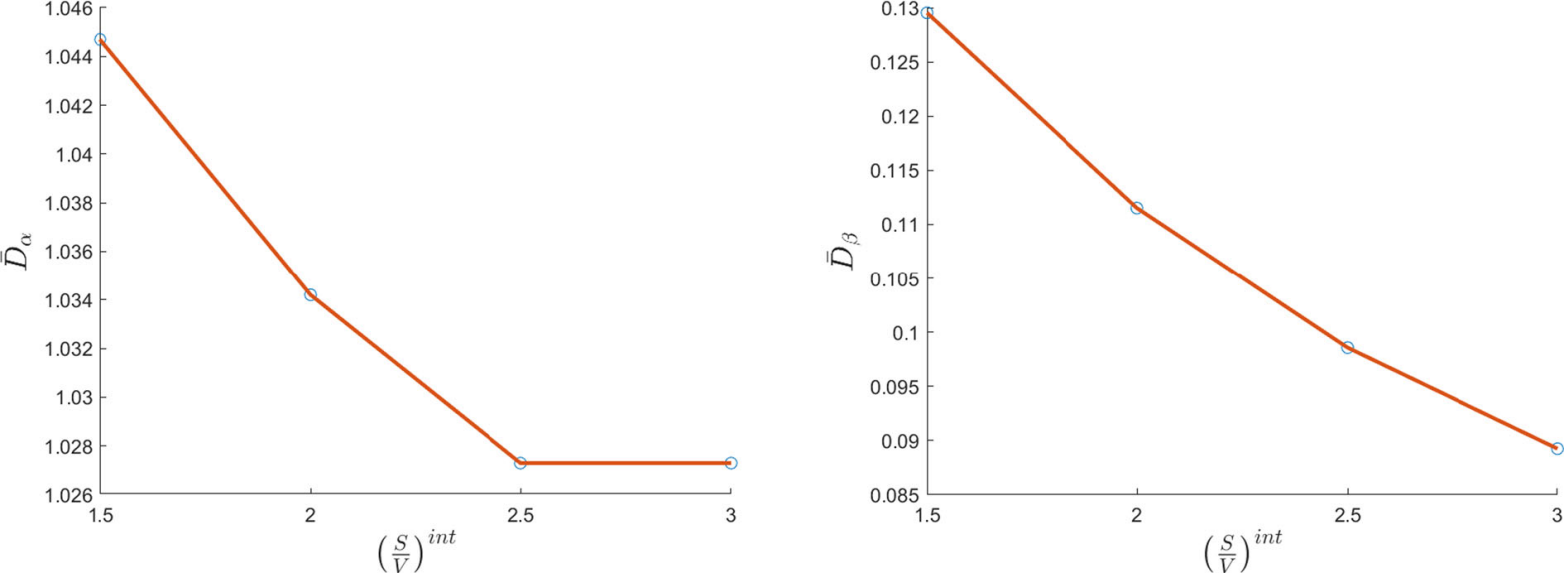
The variation of the internal diffusion coefficient $\bar{D}_\alpha$ (left plot, non-dimensional) and the external diffusion coefficient $\bar{D}_\beta$ (right plot, non-dimensional) with respect to the $(S/V)^\text {int}$ ratio (in 1/cm) varying the microstructure geometry proportion (colour figure online)

We can see a plot of the diffusion coefficient calculated by our model in Fig. 2 varying the diffusion ratio $(S/V)^\text {int}$. For every new value of $(S/V)^\text {int}$, the parameters *R* and *l* calculated by system (52) vary, leading to different microstructure geometry proportion. As we can see, we have a decreasing of both internal and external diffusion coefficients as the ratio $(S/V)^\text {int}$ increases, as expected [60]. Indeed, the ratio *S*/*V* affects the rate of water diffusion, with higher values indicating a greater number of interaction surfaces, such as cell membranes, which impede the movement of water molecules.

**Fig. 3:**
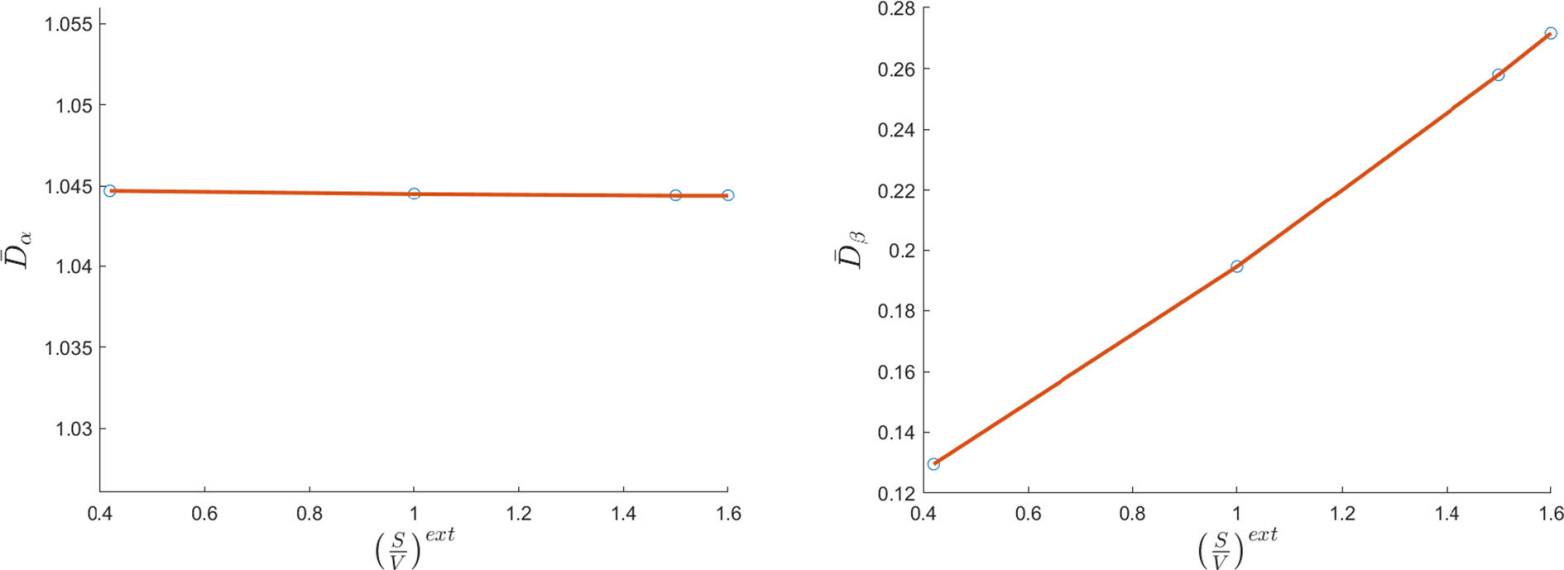
The variation of the internal diffusion coefficient $\bar{D}_\alpha$ (left plot, non-dimensional) and the external diffusion coefficient $\bar{D}_\beta$ (right plot, non-dimensional) with respect to the $(S/V)^\text {ext}$ ratio (in 1/cm) varying the microstructure geometry proportion

On the other hand, if we increase the $(S/V)^\text {ext}$ ratio we have, as expected, an opposite behavior: the external diffusion coefficient increases because the external geometry gets bigger as $(S/V)^\text {ext}$ increases. The internal diffusion coefficient remains almost constant. We can see these results in Fig. 3.

### Realistic geometry

**Fig. 4:**
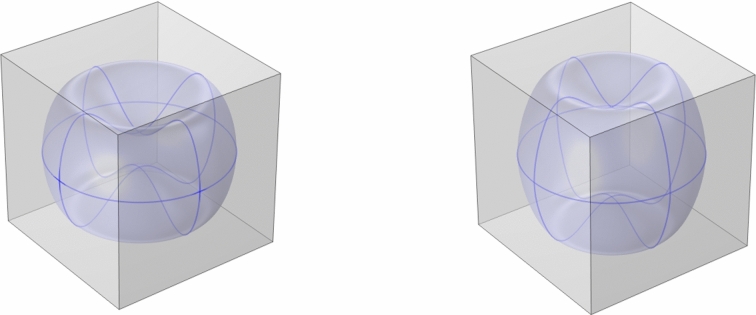
The geometry of the cell related to the cell problem with a realistic Red Blood Cell (RBC) geometry in a isotonic solution (at 300 mosmol, left) and in a hypotonic solution (at 217 mosmol, right). The blue sphere represents the erythrocytes geometry, instead the gray geometry represents the extracellular fluid geometry (colour figure online)

As the geometry of the microstructure is important to get a realistic results of the *S*/*V* ratio, a more realistic geometry of a erythrocyte can be considered. Actually, we use the following parametric expression for the erythrocytes geometry (from [22])

55 $$\begin{aligned} d(r)=\left[ 1-\left( \dfrac{r}{R_0}\right) ^2\right] ^{\frac{1}{2}}\left( C_0+C_2\left( \dfrac{r}{R_0}\right) ^2+C_4\left( \dfrac{r}{R_0}\right) ^4\right) , \end{aligned}$$

where $R_0$, $C_0$, $C_2$, and $C_4$ are fitted parameters. With the parameters of a healthy erythrocyte $R_0=3.91\,\upmu$m, $C_0=0.81\,\upmu$m, $C_2=7.83\,\upmu$m, and $C_4=-4.39\,\upmu$m, we have the same $(S/V)^\text {int}$ of above ($(S/V)^\text {int}\approx 1.5 \dfrac{1}{\mu \text {m}}$). In this situation, we find that $l=7.46\,\upmu$m, and we normalize the parameters such that $R_0'=\frac{R_0}{l}$, $C_0'=\frac{C_0}{l}$, $C_2'=\frac{C_2}{l}$, $C_4'=\frac{C_4}{l}$. We can see a plot of the geometry with these parameters in Fig. 4 (left). In this case, solving the cell problem (32), we find the following values:

56 $$\begin{aligned} \bar{D}_\alpha =\begin{bmatrix} 1.0441 & \quad 0 & \quad 0\\ 0 & \quad 1.0439 & \quad 0 \\ 0 & \quad 0 & \quad 1.0450 \\ \end{bmatrix}, \quad \bar{D}_\beta =\begin{bmatrix} 0.18551 & \quad 0 & \quad 0 \\ 0 & \quad 0.18576 & \quad 0 \\ 0 & \quad 0 & \quad 0.47038 \end{bmatrix}. \end{aligned}$$

In this case, due to the lack of symmetry in the geometry, we find a non-isotropic macroscopic diffusion coefficient with different values in the three different directions. However, given that the input microscale diffusion coefficient is symmetric, the effective diffusivity tensors which are obtained are indeed still symmetric as expected (see, e.g., [14]), as well as diagonal due to the presence of three orthogonal planes of symmetries (see also [54, 55] for recent theoretical and numerical analyses of symmetry properties of effective elliptic operators in asymptotic homogenization).

Since the erythrocyte geometry (and, in particular, the *S*/*V* ratio) is important for the determination of the macroscopic diffusion coefficient, we solve the cell problem (32) with a red blood cell in a hypotonic solution of 217 mosmol, different from the case of before (that was at 300 mosmol). We can see the geometry of this problem in the right plot of Fig. 4. In this case, the $(S/V)^\text {int}=1.16379\,\upmu$m, and the parameters are $R_0=3.8\,\upmu$m, $C_0=2.10\, \upmu$m, $C_2=7.58\,\upmu$m, and $C_4=-5.59\,\upmu$m. To maintain the same $(S/V)^\text {ext}$ as the case before, we calculate a new $l=7.59\,\upmu$m: in this way, we can focus our attention to the difference in changing only the internal *S*/*V* ratio. The results in this case are the following:

57 $$\begin{aligned} \bar{D}_\alpha =\begin{bmatrix} 1.0584 & \quad 0 & \quad 0\\ 0 & \quad 1.0584 & \quad 0 \\ 0 & \quad 0 & \quad 1.0585 \\ \end{bmatrix}, \quad \bar{D}_\beta =\begin{bmatrix} 0.24198 & \quad 0 & \quad 0 \\ 0 & \quad 0.24201 & \quad 0 \\ 0 & \quad 0 & \quad 0.28664 \end{bmatrix}. \end{aligned}$$

As we can see, the resulting diffusion coefficient (57) differs from the one found in (56). In particular, due to the fact that $(S/V)^\text {int}$ is lower in this case, we have a higher internal diffusion coefficient $\bar{D}_\alpha$. Instead, for the external diffusion coefficient $\bar{D}_\beta$, we have an increase of the value in *x* and *y* directions, and a decrease in *z* direction: this happens because the RBC geometry is more similar to a sphere than in the previous case, hence the difference of the diffusion coefficient in the three directions is lower. Due to the fact that the extracellular solution outside the cell has a lower concentration of solutes with respect to the internal of the red blood cell (hypotonic solution), water diffuses into the cell, and this explains why the internal diffusion coefficient is bigger and the norm of the external diffusion coefficient is smaller in this case.

## Conclusions

In this study, we develop and analyze a multiscale advection–diffusion model featuring both time-dependent diffusion coefficients and multiscale advective velocities with nonzero divergence, using the asymptotic homogenization technique. This framework allows us to capture the complex behavior of transport processes occurring across multiple spatial and temporal scales, particularly in systems where both diffusion and advection vary dynamically over time. By incorporating microscale time-dependence directly into the homogenization procedure, our approach extends previous models that treated the diffusion coefficient as independent of the fast temporal scale [43, 47, 48] and, by taking into account multiscale forces and the advective term, we extend the model focusing only on the diffusion equation as well [18, 34], enabling a more realistic and physically consistent description of advection–diffusion phenomena in heterogeneous media.

Taking into account the dual scales in both space and time, we have obtained the new cell problem (32), where there are derivatives with respect to both the temporal and spatial microscales. This has allowed us to incorporate a time-dependent diffusion coefficient, leading to distinct diffusion outcomes, as highlighted in Sect. 4, where we have demonstrated the differences arising from varying the time-dependent behavior of the diffusion coefficient compared to the steady-state case. At the macroscale, the diffusion coefficient is significantly different, involving a double averaging in both space and time, which results in a diffusion equation with solutions that markedly deviate from the stationary case [23, 35]. Moreover, taking into account multiscale forces and multiscale advective velocities, we obtain two additional cell problems (33) and (34), respectively. Both these cell problems depend on the microscopic time. The solutions to these cell problems lead to the emergence of additional contributions, which manifest as source terms in the resulting macroscopic Eqs. (39) and (40).

The effects of a time-dependent diffusion coefficient have been previously addressed in [18, 34], and the introduction of multiscale forces was initially explored in [56]. In this work, we have extended these earlier contributions by jointly considering the dual time-dependence of the diffusion coefficient and the influence of multiscale forcing. This has led to the formulation of two distinct cell problems, (32) and (33), which in turn contribute additional source terms in the macroscopic equation. Moreover, our model incorporates a novel source term arising from the nonzero divergence of the advective velocity field, which modifies the macroscopic advection behavior by capturing source and sink effects originating at the microscale. The presence of this term required the development of an additional cell problem, presented in (34).

The resulting macroscopic advection–diffusion equation has captured the behavior of diffusion in complex systems, offering a more accurate representation of processes influenced by temporally varying properties, such as those encountered in biological tissues. The example of water molecules in packed erythrocytes shown in the paper has demonstrated its utility in modeling biophysical phenomena where time-dependent diffusion plays a critical role [37, 42, 61].

We have applied our new model to this biological phenomenon in Sect. 5. In particular, we have demonstrated how the time-dependent diffusion coefficient can be easily incorporated into our model to determine the diffusion of water molecules in packed erythrocytes. This has allowed us to obtain different diffusion coefficients by varying the *S*/*V* ratio. The surface-to-volume ratio (*S*/*V*) is a crucial factor in understanding the water diffusion coefficient in biological tissues, as it influences diffusion rates through interactions at surfaces like cell membranes. A higher *S*/*V* ratio increases surface interactions, thereby restricting water molecule mobility.

The study of anomalous diffusion and its time-dependence is essential for understanding the microstructure of biological tissues, particularly in the brain. Diffusion Magnetic Resonance Imaging (dMRI) provides a powerful tool for investigating these phenomena. A central concept in this context is the Apparent Diffusion Coefficient (ADC), which measures the mean squared displacement of water molecules within tissues. The ADC, however, is not an intrinsic property of the tissue but is influenced by factors such as the magnetic gradient pulse sequence, including pulse duration and diffusion time [30]. ADC values in brain tissues are time-dependent, reflecting the movement constraints of water molecules and providing insights into tissue microstructure [31]. This time-dependence complicates the mathematical modeling of ADC, especially in complex diffusion regimes involving varying diffusion times, membrane permeability, and geometric configurations. Simplified models exist for short or long diffusion times (but not both), but they often rely on assumptions about tissue geometry [28]. Homogenization techniques provide a more accurate framework for modeling the time-dependence of ADC, enabling improved descriptions of complex systems. Additionally, the inverse problem in dMRI-reconstructing microstructural details from measured signals-presents significant challenges that require advanced analytical approaches [1, 16, 32, 33, 36].

The surface-to-volume ratio (*S*/*V*) is pivotal in understanding the water diffusion coefficient in biological tissues, as it governs diffusion rates through surface interactions, such as those at cell membranes. A higher *S*/*V* ratio increases surface interactions, restricting water mobility. Diffusion-weighted magnetic resonance imaging (DW-MRI) measures the apparent diffusion coefficient of water (ADCW), reflecting tissue microstructure, including cell density and membrane presence. ADCW, encapsulates water mobility across intra- and extracellular compartments, integrating structural influences like cellular membranes and extracellular tortuosity. While the ADC provides a valuable macroscopic measure, its averaging nature assumes homogeneity, complicating the interpretation of tissue microstructure [38]. Unlike classical diffusion governed by Fick’s law, ADCW accounts for these structural factors, serving as a composite metric of water mobility. We believe that our model is able to capture the time-varying ADCW, enabling precise estimation of the *S*/*V* ratio and offering valuable insights into tissue microstructure. However, additional analysis using more representative and realistic data is necessary.

Our findings highlight the crucial role of both spatial and temporal multiscale effects in modeling advection–diffusion processes, particularly in applications related to biophysics and materials science. First of all, an important direction for future work is the numerical solution of the full advection–diffusion equation, including the newly derived source terms, within realistic physiological settings. This would provide valuable insight into the model’s predictive power and practical applicability. Additionally, exploring different temporal scalings could deepen our understanding of the dual time-scale dynamics that arise in heterogeneous systems. The flexibility of the model also opens the door to further enhancements. For instance, the incorporation of complex microscale geometries, especially those extracted from medical imaging data, would allow for more accurate simulations of biological tissues. A significant extension involves coupling the diffusion of water molecules with magnetization dynamics, as relevant in diffusion magnetic resonance imaging (dMRI) [16, 32, 33], which would enhance the model’s utility in biomedical imaging contexts. Finally, our framework may be adapted to account for anomalous diffusion and its impact on the intricate temporal dynamics of biological systems, such as those found in the lymphatic system, lymph nodes, and tumor microenvironments [3, 5, 24–27]. This could ultimately lead to more effective strategies for optimizing transport and exchange processes in real-world physiological and engineered systems.

## Data Availability

There are no additional data supporting this study other than those already reported in the manuscript.
